# 

**Published:** 1875-03

**Authors:** 


### CONTENTS:

### ORIGINAL COMMUNICATIONS.

ARTICLE I.—Address by W. W. H. Thackston, M. D., D. D. S........ 481
ARTICLE II.—Proceedings of Virginia Dental Association........ 495
ARTICLE III.—Proceedings of the Georgia State Dental Society........ 503
ARTICLE IV.—Case of Resuscitation from Apparent Death from Chloroform. By A. Eubank, D. D. S........ 505

### SELECTED ARTICLES.

ARTICLE V.—Comments on the Recent Address of Dr. Allport, before the Boston Academy of Dental Science. By J. Richardson, D. D. S........ 506
ARTICLE VI.—Precautions During Anæsthesia. By Dr. Bigelow........ 513
ARTICLE VII.—Lancing the Gums. By Dr. James Finlayson........ 516

### EDITORIAL, ETC.

The Thirty-Fifth Annual Commencement of the Baltimore College of Dental Surgery..... 518

### BIBLIOGRAPHICAL.

Dental Pathology and Surgery. By S. James Salter, M. B., F. R. S........ 521

The Treatment of Pleurisy. By Jno. W. Côrson, M. D........ 522

### OBITUARY.

Eleazar Parmley, M. D., D. D. S........ 522

Asa Hill, D. D. S........ 523

George E. Hawes, D. D. S........ 523

James Fleming, M. D., D. D. S........ 524

### MONTHLY SUMMARY.

Supplemental Nerve Force........ 524
False Tongue........ 525
New Sign of Death........ 525
Treatment of Epistaxis........ 525
Danger of Chloroform after Chloral........ 525
An Insecticide........ 525
Preservation of Leeches........ 526
Chloral as a Preservative........ 526
New Operation for Cleft Palate and Blind Uvula........ 527
Malignant Pustule from a Fly-bite........ 527
Lock-jaw and Quinia........ 528
Decision Affecting Dentistry........ 528
Precautions Against Trichina........ 528

